# The utility of an artificial intelligence model based on decision tree and evolution algorithm to evaluate steatotic liver disease in a primary care setting

**DOI:** 10.1590/1414-431X2025e14538

**Published:** 2025-06-16

**Authors:** A.C. Goulart, A.P. Alencar, G. Tunes, L.L.T. Bianchi, M.H. Miname, C.M. Padilha, J.M.S. Pescuma, A.L.C.C. Rodrigues, B.B. Henares, M.S. de Almeida, T.A.O. Machado, D.H. Syllos, Y.P. Wang, M. Rienzo

**Affiliations:** 1 Centro de Acompanhamento da Saúde e Check-up, Hospital Sírio-Libanês, São Paulo, SP, Brasil; 2 Departamento de Epidemiologia, Faculdade de Saúde Pública, Universidade de São Paulo, São Paulo, SP, Brasil; 3 Instituto de Matemática e Estatística, Universidade de São Paulo, São Paulo, SP, Brasil; 4 Departamento de Psiquiatria, Faculdade de Medicina, Universidade de São Paulo, São Paulo, SP, Brasil

**Keywords:** Steatotic liver disease, Decision analysis, Primary care, Screening

## Abstract

Many ways of classifying steatotic liver disease (SLD) with metabolic conditions have been proposed. Thus, SLD-related variables were verified using a decision tree. We tested if the suggested components of the actual classification (metabolic dysfunction-associated steatotic liver disease, MASLD) are also present in young and middle-aged adults. In a cross-sectional study involving 6,839 adults (median age: 46 years, 69.5% men) in a primary care setting, a decision tree was created to determine potential clinical and laboratory variables related to SLD. The odds ratio (OR) with a respective 95% confidence interval (95%CI) was calculated for both sexes. SLD frequency was 26.6% (23% in men). More variables and with higher ORs for the association with SLD were identified in women: category 1 (body mass index (BMI) ≥29 kg/m^2^, age <51 years, high-sensitivity C-reactive protein (hs-CRP) ≥0.195 mg/dL): OR=10.9, 95%CI: 4.40-26.6; category 2 (BMI <9 kg/m^2^, metabolic syndrome (MS), age ≥50 years, neck circumference (NC) ≥36 cm): OR=8.1, 95%CI: 2.2-29.9; and category 3 (BMI ≥29 kg/m^2^, age <51 y-old, dyslipidemia, high-density lipoprotein cholesterol (HDL-c) <42 mg/dL): OR=4.7, 95%CI: 2.20-10.7. For men: category 1 (waist circumference (WC) ≥101 cm, alanine aminotransferase (ALT) <28 mg/dL, glycated hemoglobin (HbA1c) ≥5.7%): OR=4.7, 95%CI: 2.8-7.9; and category 2 (WC ≥101 cm, ALT ≥28 mg/dL): OR=3.2, 95%CI: 2.5-4.0). The decision tree identified more variables related to SLD, particularly in women, such as age of more than 50 years, elevated hs-CRP, and NC≥36 cm than variables related to MASLD.

## Introduction

The progressive increase in the prevalence of steatotic liver disease (SLD) by more than 100% with a concomitant escalation in non-alcoholic steatohepatitis (NASH), now named metabolic dysfunction-associated steatohepatitis (MASH) (1), makes this clinical condition a new challenge for public health policies (2,3). In fact, SLD is one of the five top leading causes of death due to cirrhosis, contributing to chronic disease burden, particularly in individuals with cardiometabolic conditions such as obesity, glucose metabolism dysregulation, dyslipidemia, and hypertension (4).

In addition to the direct hepatic damage, steatosis can turn into cirrhosis and be associated with hepatocarcinoma risk (3,5) and many other extrahepatic manifestations, including increased risk of metabolic conditions, cardiovascular disease (CVD), depression, and finally mortality (6-10). With the progressive increase of the prevalence and complications related to SLD, a more efficient way to identify predictors is needed, improving treatment outcomes. In 2020, a consensus of international experts proposed a new definition for SLD associated with metabolic dysfunction (metabolic dysfunction-associated fatty liver disease, MAFLD) (11), which identifies subjects at higher risk of hepatic or cardiovascular non-fatal and fatal outcomes better than the condition previously denominated non-alcoholic fatty liver disease (NAFLD) (8-10). Some recent data, however, have led to controversial findings about the importance and prognostic value of a broader metabolic definition, but at the same time ignoring other liver disease etiologies with an important impact on prognosis, such as excessive alcohol intake and viral hepatitis (12-17). In 2023, the Multinational Liver Societies proposed the nomenclature metabolic dysfunction-associated alcoholic liver disease (MetALD) for patients with MASLD who consume higher amounts of alcohol per week (140 and 210 g/week for females and males, respectively). In addition, cryptogenic SLD is used for individuals with no metabolic parameters and unknown origin. Also, metabolic dysfunction-associated steatohepatitis (MASH) replaced NASH (18). According to National Health and Nutrition Examination Survey (NAHNES) 2017-2020, among patients with SLD (37.87%), most were classified as having MASLD (32.45%), followed by the other categories: MetALD (2.56%), alcoholic liver disease (ALD) (1.17%), and other causes (0.32%) among young-to-middle-aged adults (19). These population findings are troubling and reinforce the importance of better identifying and treating potential metabolic conditions among younger individuals of a productive age to prevent SLD complications. Thus, we tested additional variables that could be associated with SLD using a decision tree to verify if the suggested components of the current classification (MASLD) can also be used in young and middle-aged adults (both sexes) or if more variables would emerge in a Brazilian primary care setting.

## Material and Methods

### Population and study design

This cross-sectional study investigated SLD-related variables using decision tree regression in apparently healthy adults. Participants were consecutively recruited from the Health Promotion and Check-up Center, a branch of a complex of health facilities of the Hospital Sírio-Libanês (HSL), located in the city of São Paulo, Brazil. An international benchmark in healthcare, HSL has been providing patient care and innovation in education and research for nearly a century. Every year, the hospital treats thousands of patients under preventive medicine programs and treats urgent and emergency cases, providing high-complexity therapeutic treatments and rehabilitation care, among others. Approximately 300 adults seek medical appointments in the Health Promotion and Check-up Center for prevention and routine check-up exams monthly. The institutional protocol of the Check-up Center is based on international guidelines for promoting health and prevention for the adult population (https://www.uspreventiveservicestaskforce.org/uspstf/).

All adults older than 18 years of age who could understand and complete the research questionnaire by themselves and had sought the outpatient unit at the Health Promotion and Check-up Center for prevention purposes were invited to participate in the present study. Participants answered a standardized questionnaire that included sociodemographic and clinical information, screening scales on mental health, and anthropometric and blood pressure measurements. Also, an abdominal ultrasound was performed to screen for SLD.

Individuals with acute or chronic active hepatitis based on serological status or active liver diseases at admission were excluded from the current analyses. From 7,241 patients who agreed to participate in the study between January 2018 and August 2020, 402 individuals who reported alcohol consumption suggestive of abuse or dependence and, thus, had an ALD diagnosis were excluded. In the end, 6,839 adults were considered for the present analyses. The participation rate was extremely high, with 99.9% of invited attendees accepting to take part in the current study.

### Definition of SLD

In the present study, the definition of SLD was based on the exclusion of ALD and other chronic liver conditions such as B or C virus hepatitis (HBV and HCV) and showing diffuse hepatic fatty infiltration on the B-mode ultrasound imaging based on the following criteria: 1) parenchymal brightness; 2) deep-beam attenuation; 3) vascular blurring (loss of echoes from the walls of the portal veins); and 4) increasing discrepancy of echogenicity between the liver and kidney parenchyma (hepatorenal index) (20-22).

We considered alcohol consumption above 210 g/week for men or 140 g/week for women, as well as the Alcohol Use Disorders Identification Test (AUDIT) (23) scoring (hazardous or harmful alcohol consumption (8 to 14 points) and alcohol dependence (15 or more points)) for the classification of ALD. All the participants with hepatic steatosis, according to the abovementioned ultrasound criteria, who scored lower than 8 (low-risk consumption) in AUDIT, and with alcohol intake below 210 or 140 g/week for men and women, respectively, were classified as having SLD.

### Procedures

During the participant’s regular multidisciplinary appointment on a typical weekday, a research assistant approached consecutive attendees in the waiting room and invited them to participate. An experienced nutritionist measured participants' height, weight, and waist circumference, and a nurse measured blood pressure and collected ancillary exams. A team of previously trained medical assistants was responsible for collecting information about medical diagnoses and medication use.

### Sociodemographic and clinical variables

Sociodemographic data included age, sex, marital status, educational level, occupational status, and tobacco use. Previous medical diagnoses or medication use were self-reported by attendees and confirmed by clinical examinations performed in a primary care setting.

### Anthropometric and adiposity measures

Anthropometric measurements were duplicated according to the National Health and Nutrition Examination Survey (NHANES) III procedures (24). In cases where the first two measures differed by more than 0.5 cm, a third measure was recorded, and the average of all measures was computed. Body weight (0.2 kg increments), body fat percent (BF%) (0.1% increments), and lean mass (kg) were measured by a bioelectrical impedance analysis (BIA) technology using InBody370S (Ottoboni Ltda., Brazil), as well as height was measured in meters using a portable stadiometer (Seca Corporation, USA) to calculate body mass index (BMI) (kg/m^2^). Circumferences were measured with Gulick tape. Waist circumference was measured at the umbilicus and recorded to the nearest 0.1 cm. Neck circumference (NC) was measured below the laryngeal prominence and perpendicular to the long axis of the neck, and the minimum circumference was recorded to the nearest 0.1 cm (25). The NC measure was internally (Check Center) evaluated, and the average Pearson’s inter-examiner correlation coefficient for NC was 0.99, showing excellent reproducibility.

### Cardiometabolic risk factors

Diagnoses of dyslipidemia and metabolic syndrome (MS) were based on definitions of the National Cholesterol Program-Adult Treatment Panel III (NCEP ATP III): for dyslipidemia: low density lipoprotein (LDL) cholesterol ≥130 mg/dL and/or lipid-lowering drugs and for MS: three or more criteria including waist circumference (WC) >88 cm for women or >102 cm for men, HDL cholesterol <50 mg/dL for women or <40 mg/dL for men, a systolic blood pressure (SBP) ≥130 or diastolic blood pressure (DBP) ≥85 mmHg, serum triglyceride levels ≥150 mg/dL, and fasting plasma glucose ≥110 mg/dL (26).

Participants were classified as having high blood pressure if they had SBP≥140 mmHg or diastolic blood pressure (DBP)≥90 mmHg or reported antihypertensive drug treatment.

Diabetes was defined as a medical history of diabetes, use of medication to treat diabetes, or fasting plasma glucose ≥126 mg/dL or glycated hemoglobin (HbA1c) ≥6.5% (26).

Individuals were classified as “insufficiently active” if they reported <150 min per week of moderate physical activity or <75 min per week of intense physical activity in the leisure domain. Smoking status was classified as never, past, or current smoker.

### Ancillary exams

A 12-h fasting venous blood sample was obtained for the biochemical analysis of the main biomarkers: fasting glucose levels, HbA1c, total cholesterol and its fractions, triglycerides, high-sensitivity C-reactive protein (hs-CRP), aspartate aminotransferase (AST), alanine aminotransferase (ALT), and gamma-glutamyl transferase (Gamma GT).

### Statistical analysis

Baseline characteristics are reported as percentages (%) for categorical variables and means±SD or medians with interquartile ranges (IQRs) for continuous variables according to their distribution evaluated by the Kolmogorov-Smirnov normality test. Chi-squared and Student’s *t*-test or Mann-Whitney test were used to compare main descriptive characteristics according to SLD stratified by sex.

A regression tree model (27) was fitted to classify everyone with or without SLD. Each branch identifies an explanatory variable (from all variables collected) that discriminates SLD. For quantitative variables, the regression tree finds threshold values to classify individuals. Individuals with values larger or lower than this threshold had a higher or lower probability of having SLD. These variables and thresholds are chosen to minimize the classification error in the regression tree. After building the tree, the percentage of steatosis cases was identified for each tree node. Each final node defines categories that take into consideration all chosen variables. A logistic regression model was fitted to test which final nodes had a significant effect on predicting steatosis. The odds ratio (OR) was calculated for each predictive model with the respective 95% confidence interval (95%CI). All analyses were performed in males and females separately using the free software R (library *Rpart*) and SPSS version 25.0 (IBM, USA).

## Results

The main baseline characteristics of the 6,839 participants are shown in Table 1. In brief, the median age was 46 years, (IQR: 41-54 years), most were men (69.5%), White (93.2%), married (77.7%), and with a high level of education (college or higher: 96.6%). The overall frequency of SLD detected by routine ultrasound was 26.6% (23% in men). SLD patients had higher frequencies of metabolic risk factors such as obesity, high adiposity and anthropometric indexes, metabolic syndrome (MS), diabetes, dyslipidemia, and hypertension than non-SLD participants. In addition, individuals with SLD had poorer lifestyle habits (sedentarism and smoking).

**Table 1:** Baseline characteristics of 6,839 apparently healthy adults from a Brazilian check-up center, according to the presence or absence of steatotic liver disease (SLD).

	SLD		Total (n=6,839)	P-value
	No (n=5,018)	Yes (n=1,821)		
Age, median years	43 (37-49)	49 (42-55)	46 (41-54)	<0.0001
Sex				
Men	3,188 (63.5)	1,570 (86.2)	4,758 (69.6)	<0.0001
Women	1,830 (36.5)	251 (13.8)	2,081 (30.4)	
Race				0.43
White	4,246 (93.3)	1,543 (92.7)	5,789 (93.2)	
Brown	178 (3.9)	63 (3.8)	241 (3.9)	
Black	48 (1.1)	19 (1.1)	67 (1.1)	
Yellow	78 (1.7)	39 (2.3)	117 (1.9)	
Educational level				<0.0001
Without education	29 (0.6)	8 (0.4)	37 (0.5)	
Elementary	11 (0.2)	17 (0.9)	28 (0.4)	
High School	92 (1.8)	65 (3.6)	157 (2.3)	
College or above	4,848 (97.4)	1,722 (95.0)	6,570 (96.6)	
Marital status				<0.0001
Married	3,756 (75.3)	1,524 (84.2)	5,280 (77.7)	
Single	866 (17.4)	169 (9.3)	1,035 (15.2)	
Divorced	329 (6.6)	103 (5.7)	432 (6.4)	
Windowed	35 (0.7)	15 (0.8)	50 (0.7)	
Smoking				<0.0001
Never smoker	4,458 (89.2)	1,522 (84.1)	5,980 (87.8)	
Past smoker	1 (0.0001)	0	1 (0.001)	
Current smoker	539 (10.8)	288 (15.9)	827 (12.1)	
Insufficiently active	2,071 (41.5)	1,111 (61.3)	3,182 (46.8)	<0.0001
Metabolic syndrome	368 (7.3)	730 (40.1)	1,098 (16.1)	<0.0001
Hypertension	1,061 (21.1)	738 (40.5)	1,799 (26.3)	<0.0001
Diabetes	68 (1.4)	161 (8.8)	229 (3.3)	<0.0001
Dyslipidemia	2,192 (43.7)	1,397 (76.7)	3,589 (52.5)	<0.0001
Obesity	473 (9.5)	825 (46.1)	1,298 (19.1)	<0.0001
BMI, median (kg/m^2^)	25.4 (23.4-27.6)	29.6 (27.4-32.3)	26.8 (24.6-29.6)	<0.0001
Fat body (%)	26.0 (20.9-32.3)	31.2 (26.4-36.1)	27.7 (22.5-33.9)	<0.0001
Waist circumference (cm)	91.5 (84.0-98.0)	104.0 (98.0-111.0)	96.0 (88.0-104.0)	<0.0001
Total cholesterol (mg/dL)	184 (162-207)	185 (162-212)	188 (165-212)	<0.0001
LDL-cholesterol (mg/dL)	108 (88-130)	115 (92-137)	113 (93-136)	<0.0001
HDL-cholesterol (mg/dL)	52 (44-63)	42 (37-50)	48 (40-59)	<0.0001
Triglycerides (mg/dL)	95 (75-128)	149 (107-204)	113 (82-162)	<0.0001
Fasting glucose (mg/dL)	91 (86-96)	97 (91-104)	93 (88-99)	<0.0001
HbA1c (%)	5.1 (4.9-5.3)	5.3 (5.1-5.6)	5.2 (5.0-5.5)	<0.0001
hs-CRP (mg/dL)	0.10 (0.05-0.20)	0.16 (0.09-0.35)	0.11 (0.06-0.23)	<0.0001
AST (mg/dL)	19 (16-23)	22 (18-27)	20 (17-24)	<0.0001
ALT (mg/dL)	20 (15-27)	31 (23-42)	23 (17-32)	<0.0001
Gamma GT (mg/dL)	18 (12-26)	29 (21-44)	21 (15-33)	<0.0001

Hypertension was defined as systolic blood pressure ≥140 mmHg or diastolic blood pressure ≥90 mmHg. History of hypertension was diagnosed by a physician or by current treatment. Dyslipidemia was defined according to guidelines of the National Cholesterol Program-Adult Treatment Panel III (NCP-ATPIII, ref.) as follows: LDL-cholesterol ≥130mg/dL or use of lipid lowering drug. Diabetes was defined as previous medical history of diabetes, use of medication to treat diabetes, a fasting plasma glucose ≥126 mg/dL, a 2-h plasma glucose ≥200 mg/dL, or HbA1c ≥6.5%. BMI: body mass index; Obesity: BMI ≥30kg/m^2^. Metabolic syndrome was defined according to NCEP ATP III (ref.). LDL: low-density lipoprotein; HDL: High density lipoprotein; HbA1c: glycated hemoglobin; hs-CRP: high-sensitivity C-reactive protein; ALT: alanine transaminase; AST: aspartate transaminase; Gamma GT: gamma glutamyl transferase. Data are reported as median (IQR: interquartile range) or number (%); P-values were obtained by the Chi-Squared test or Mann-Whitney test.

**Figure 1:**
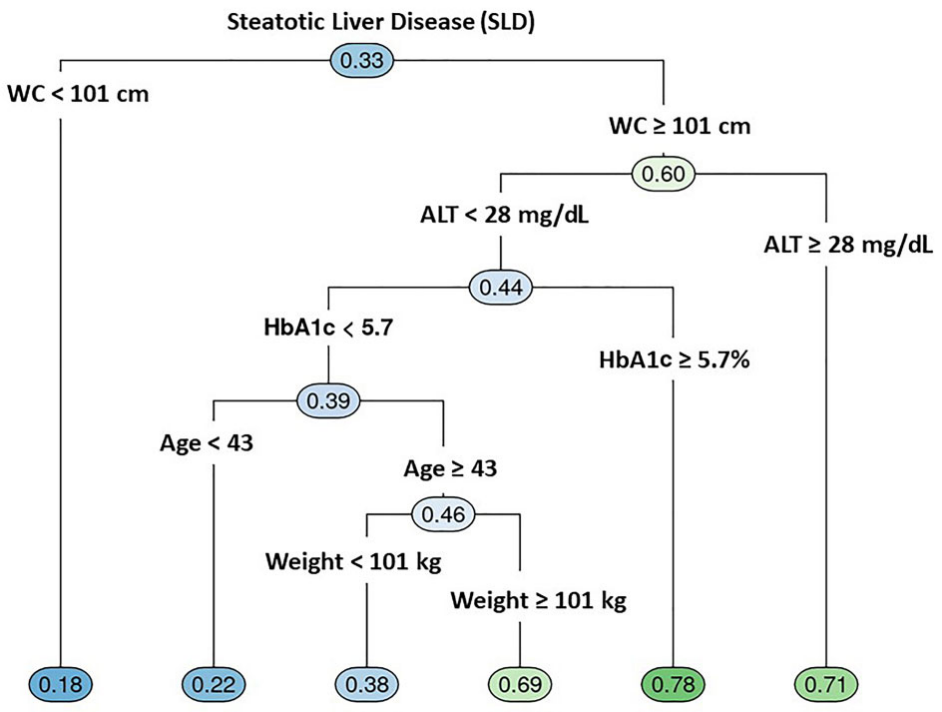
Decision tree for determination of steatotic liver disease (SLD) predictors among 4,758 men. WC: waist circumference; ALT: alanine aminotransferase; HbA1c: glycated hemoglobin.

**Figure 2:**
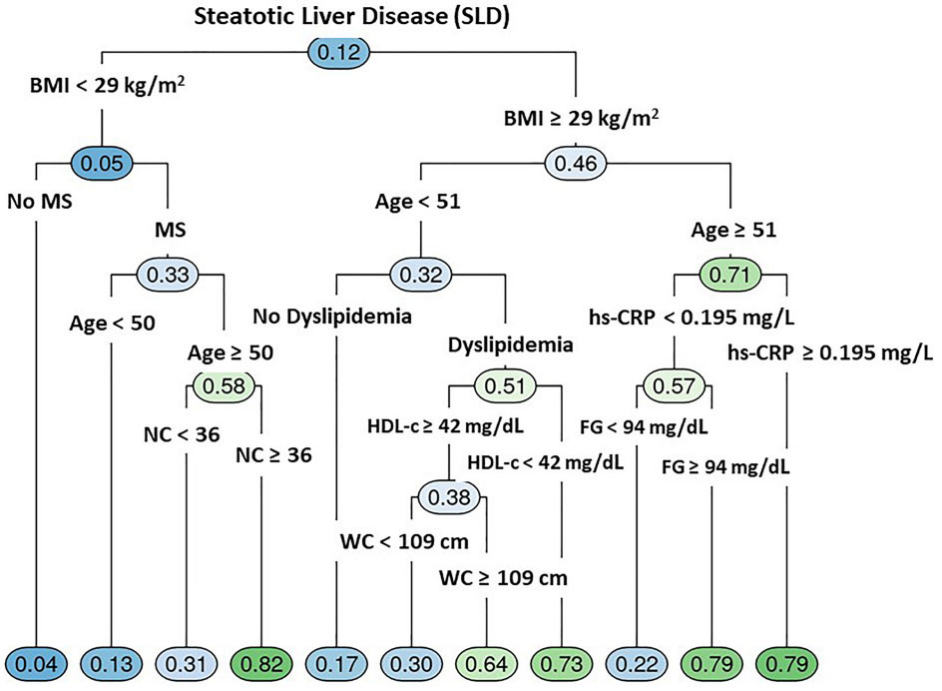
Decision tree for determination of steatotic liver disease (SLD) predictors among 2,081 women. BMI: body mass index; MS: metabolic syndrome; NC: neck circumference; HDL-c: high-density lipoprotein cholesterol; hs-CRP: high-sensitivity C-reactive protein: FG: fasting glucose; WC: waist circumference.

The decision trees for determining SLD-related variables among men and women are shown in Figures 1 and 2. In men, the frequency of SLD was 33%, while in women it was much lower (12%). Despite the higher frequency of SLD in men, women presented more variables (possible predictors) with higher ORs related to SLD. For women, the first variable selected was BMI≥29 kg/m^2^ followed by age ≥50, NC ≥36 cm, hs-CRP ≥0.2 mg/L, fasting glucose ≥94 mg/dL, HDL-c <42 mg/dL, dyslipidemia, and WC ≥109 cm. For men, WC ≥101 cm was the first variable related to SLD selected in the tree, followed by HbA1c ≥5.7%, ALT ≥28, and weight ≥101 kg.

Table 2 shows the main categories with the selected SLD-related variables for women and men according to the decision tree regression.

**Table 2:** Predictors of steatotic liver disease (SLD) for men and women according to decision tree: a comparison of logistic regression models.

Categories	Predicted factors	OR (95%CI)	P-value
Men (n=4,758)			
Category 1	WC ≥ 101 cm, ALT < 28 mg/dL, HbA1c ≥ 5.7%	4.7 (2.8-7.9)	<0.0001
Category 2	WC ≥ 101 cm, ALT ≥ 28 mg/dL	3.2 (2.5-4.0)	0.0004
Category 3	WC ≥ 101 cm, ALT < 28 mg/dL, HbA1c < 5.7%, age < 43 y-old	0.4 (0.2-0.5)	<0.0001
Category 4	WC < 101 cm	0.3 (0.2-0.3)	0.0015
Women (n=2,081)			
Category 1	BMI ≥ 29 kg/m^2^, age < 51 y-old, hs-CRP ≥ 0.195 mg/dL	10.9 (4.40-26.6)	<0.0001
Category 2	BMI < 29 kg/m^2^, MS, age ≥ 50 y-old, NC ≥ 36 cm	8.1 (2.2-29.9)	<0.0001
Category 3	BMI ≥ 29 kg/m^2^, age < 51 y-old, dyslipidemia, HDL-c < 42 mg/dL	4.7 (2.0-10.7)	<0.0001
Category 4	BMI ≥ 29 kg/m^2^, Age < 51 y-old, without dyslipidemia	0.4 (0.2-0.7)	0.0004
Category 5	BMI < 29 kg/m^2^, MS, Age < 50 y-old	0.3 (0.1-0.7)	<0.0001
Category 6	BMI < 29 kg/m^2^, without MS	0.1 (0.0-0.1)	0.0015

WC: waist circumference; ALT: alanine aminotransferase; HbA1c: glycated hemoglobin; BMI: body mass index; hs-CRP: high-sensitivity C-reactive protein; MS: metabolic syndrome; NC: neck circumference; OR: odds ratio; 95%CI: confidence interval.

## Discussion

Based on the decision tree regression, there were more variables that indicated clinical suspicion of SLD in women (BMI ≥29 kg/m^2^, age ≥50 y, dyslipidemia, MS, hs-CRP ≥0.195, low HDL-c <42, and NC ≥36 cm) than in men (waist circumference ≥101, ALT ≥28, HbA1c ≥5.7%). These findings suggest the potential value of feasible measurements commonly used in clinical practice to screen for SLD early and consequently prevent a potentially poor prognosis.

Some metabolic abnormalities defined as significant components of steatosis in the MAFLD definition (11) (overweight/obesity, type 2 diabetes mellitus, or for lean/normal weight individuals with at least two metabolic factors such as WC ≥102/88 for men and women, respectively, BP ≥130/85 mmHg dysregulation, regardless of the etiology of liver disease) were also identified in our tree regression model, particularly among women.

Our study was similar to that of Etminani et al. (12) who evaluated the metabolic profile for predicting SLD diagnosed by ultrasound in a sample of 413 adults aged 30-60 years (60.5% women) without alcohol abuse (less than two drinks/day) and hepatitis B or C. The overall prevalence of ultrasound-diagnosed SLD was higher than in our study (39.3%). SLD diagnosis was also more frequent among men than in women (42.3 *vs* 30.4%; P<0.05). Overall, male gender, BMI ≥25 kg/m^2^, ALT ≥41 IU/I, fasting glucose >100 mg/dL, and high ferritin (≥248 ng/mL in men and ≥150 ng/mL in women) were identified as likely predictors of SLD. The only significant factors associated with SLD among men were high BMI (OR=6.146; 95%CI: 2.183 17.299) and high ALT (OR=3.294; 95%CI: 1.288-8.425). Among women, high BMI (OR: 5.952; 95%CI: 1.751-20.226), high fasting glucose (OR: 2.925, 95%CI:1.343-6.370), and high ferritin (OR=3.737; 95%CI: 1.235-11.308) were significantly associated factors (P<0.05 for all variables) (12). Although we did not include ferritin in our analyses, there were some similarities with our study regarding predictors of SLD, such as BMI and ALT. However, the cut-off values of the predictors used in the regression tree differed, and a wider group of SLD-related variables were selected in the risk model. This strategy is more reliable since the decision tree (27) methodology discriminates the main variables with their respective cut-offs, which are more associated with SLD for men and women.

Our study found additional metabolic factors other than liver diseases, revealing a different perspective for evaluating the risk of future complications compared to previous studies that used the MAFLD definition to investigate hepatic and extrahepatic complications and mortality (8-10). It seems that the most valuable role of MAFLD is in discriminating more advanced complications in the liver such as fibrosis (10). The meta-analysis of Ayada et al. (10) included 17 studies comprising 9,808,677 individuals from the general and patient population to investigate differences between MAFLD and NAFLD. In the general population, the MAFLD-only group was associated with a significantly increased risk of fibrosis (relative risk (RR): 4.2; 95%CI: 1.3-12.9) and had higher ALT (mean difference: 8.0 U/L, 95%CI: 2.6-13.5) and AST (mean difference: 6.4 U/L, 95%CI: 3.0-9.7), compared to NAFLD-only.

Additional data from NHANES III (n=12,878) and NHANES 2017-2018 (n=43,280) on fatty liver disease (FLD) by ultrasound were used to investigate mortality. In this population-based data, MAFLD and NAFLD had similar clinical profiles and long-term outcomes. The higher liver-related mortality among those with NAFLD was due to insulin resistance, while MAFLD was primarily detected in individuals with ALD (10). A Brazilian retrospective study based on liver biopsy reported both NAFLD and MAFLD definitions with intermediate/high 10-year cardiovascular risk (CVR) as well as a high CVD rate. Patients with MAFLD and concomitant viral infection showed significantly increased 10-year CVR and CVD compared to those without viral infection (15). This finding reinforces the importance of not ignoring the etiology of liver damage because of the additional mortality risk beyond that posed by metabolic risk factors. Another important aspect is the emphasis on metabolic dysfunction alone, underestimating the impact of steatosis itself on fatty liver disease. Non-metabolic risk (MR)-SLD individuals, although few, can have severe hepatic steatosis with significant liver injury and fibrosis and, thus, more attention should be given to this population in clinical practice (14). Therefore, it is particularly important to propose different ways to discriminate potential and different SLD-related variables by sex, such as neck circumference and hs-CRP, as we explored with the decision tree.

The decision tree estimates a threshold value for each laboratory parameter to minimize classification error among those with or without steatosis. Our upper limit for hs-CRP was 0.195 mg/L for women whose BMI was 29 kg/m^2^ or higher who were 50 years old or older. This hs-CRP cut-off is in the intermediate CVR range (0.1 to 0.3 mg/dL) according to the laboratory methodology applied (immunoturbidimetry). Thus, the hs-CRP threshold for the women’s decision tree detected by our laboratory (0.195 mg/dL) might correspond to an increased CVR in this subset of participants.

### Strengths

The regression tree strategy is a valuable tool for finding several variables and their thresholds for SLD simultaneously. The assessment of steatosis by the decision tree is based on the proportions of steatosis corresponding to each branch of the tree. Moreover, the present decision tree algorithm identified more and different variables related to SLD than those reported in the current definition of MASLD. It is worth noting that the regression tree can consider the interaction effects that are naturally incorporated into medical practice. We identified different prediction patterns for each sex that have not been reported in any previous study. Thus, the present study presents a valuable tool that uses feasible variables that can be easily investigated in clinical practice in the population with SLD.

### Limitations

First, although the study sample is large, it is not representative of the general Brazilian population, as the data were from young and middle-aged individuals who sought care at Hospital Sírio-Libanês, a high-end private healthcare facility. Nevertheless, we could identify a high rate of SLD associated with many clinical and laboratory factors, which raised the possibility of more severe SLD. Future studies in different segments of the health system and with diverse socioeconomic backgrounds should be conducted to confirm our findings.

Second, as the recruitment was non-probabilistic, the sample cannot be considered representative of the population attending primary care.

Third, the cross-sectional design of the study does not allow any conclusive statements to be made about causality. Reverse causality commonly emerges in cross-sectional studies, where residual confounding might remain even after adjustment. However, an interesting hypothesis might emerge under the regression tree methodology. Variables can operate through a common etiological factor or in conjunction with the causal chain of progression to cirrhosis.

Fourth, SLD diagnosis was evaluated as a binary variable (yes/no) without considering the severity of SLD, which could result in a different selection of variables.

Finally, we had more than 20% missing values for ferritin, making it impossible to consider this variable in the regression models.

## Conclusions

More clinical and laboratory variables, considering interaction effects with higher ORs related to SLD, were detected by the decision tree in women than in men. The decision tree found more factors associated with SLD, particularly in women, such as age more than 50 years, hs-CRP ≥0.195 mg/dL, and NC ≥36 cm than those related to MASLD. The findings suggest that routine screening with feasible and simple measurements often used in primary care might be helpful in diagnosing SLD.
